# *De novo* subgaleal abscess – a rare presentation of melioidosis: a case report

**DOI:** 10.1186/s13256-018-1643-x

**Published:** 2018-04-30

**Authors:** Chamara Dalugama, Asanka Tennegedara, Indika Bandara Gawarammana

**Affiliations:** 1 Department of Medicine, University of Peradeniya, Peradeniya, Sri Lanka; 2 Department of Microbiology, University of Peradeniya, Peradeniya, Sri Lanka; 3 Department of Medicine, University of Peradeniya, Peradeniya, Sri Lanka

**Keywords:** *Burkholderia pseudomallei*, Melioidosis, Subgaleal abscess, Sri Lanka

## Abstract

**Background:**

Melioidosis is an emerging infection in the tropics caused by the bacterium *Burkholderia pseudomallei.* Poorly controlled diabetes is a known risk factor. Melioidosis has a broad spectrum of clinical manifestations ranging from a localized abscess to pneumonia to disseminated sepsis with multiorgan failure. Pyrexia of unknown origin is a common presentation. Abscesses in unusual anatomical locations are well known to be associated with melioidosis.

**Case presentation:**

We report a case of a 64-year-old Sri Lankan Sinhalese man with prolonged fever and constitutional symptoms with a neglected swelling over the back of the scalp who was found to have an abscess in the subgaleal space of the scalp during surgical drainage. *Burkholderia pseudomallei* was isolated in pus culture, and melioidosis serology was highly positive. The patient was treated with ceftazidime for 2 weeks, followed by co-trimoxazole for another 3 months. He made a complete clinical recovery with normalization of inflammatory markers. To the best of our knowledge, this is the first case of subgaleal abscess following melioidosis infection reported in the literature.

**Conclusions:**

Abscesses in anatomically unusual locations should raise suspicion for melioidosis infection, particularly among patients with risk factors such as diabetes mellitus.

## Background

Pyrexia of unknown origin (PUO) is a commonly encountered difficult diagnostic problem in clinical medicine [1]. Melioidosis is an emerging infection caused by *Burkholderia pseudomallei,* which is a gram-negative rod found in soil and water [2–5]. Melioidosis is an emerging infection predominantly in the tropics, including Sri Lanka, and the risk factors associated with it are diabetes, heavy alcohol use, chronic pulmonary disease, chronic renal disease, thalassemia, glucocorticoid therapy, and cancer [6]. It can have protean clinical manifestations at its presentation, including PUO [7–12]. Diagnosis of melioidosis requires a high degree of clinical suspicion and the availability of laboratory facilities. Early and correct diagnosis and institution of proper antimicrobial therapy are important to reducing morbidity and mortality. We present what is, to the best of our knowledge, the first reported case of a *de novo* subgaleal abscess due to melioidosis, followed by complete recovery with treatment.

## Case presentation

We report a case of a 64-year-old Sri Lankan Sinhalese man from North Central Province referred to Peradeniya Teaching Hospital with a history of fever of 1 month’s duration. He was a paddy farmer by profession. He had had type 2 diabetes mellitus for 10 years with poor glycemic control and was receiving mixtard insulin.

He complained of generalized malaise and anorexia of 1 month’s duration with daily fever spikes. He was having chills with drenching night sweats. He complained of severe anorexia and had weight loss of 6 kg over the last month. He denied having long-standing cough, urinary symptoms, or alteration of bowel habits. He had no past history or contact history of tuberculosis. He complained of a low-grade headache at the back of the head. He had no photophobia or phonophobia.

On physical examination, he was observed to be wasted and ill-looking. He was moderately pale and not icteric. He did not have features of meningism. He did not have cervical or axillary lymphadenopathy. His pulse rate was 84 beats per minute, and his blood pressure was 130/80 mmHg. A precordium examination did not reveal any murmurs. Respiratory and abdominal examinations was unremarkable. The results of a neurological examination were normal with nonproliferative diabetic retinopathy in both fundi. At the back of the scalp, the patient had boggy swelling with dimensions of 3 × 3 cm that was fluctuant and tender on palpation.

The patient’s complete blood count showed hemoglobin of 7.8 g/dl with mean corpuscular volume of 80 fl, platelet count of 470 × 10^3^/μl, and white blood cell count of 7.7 × 10^6^/μl. His C-reactive protein (CRP) level was 141 IU/L, and his erythrocyte sedimentation rate (ESR) was 140 mm in the first hour. His transaminases and fractionated bilirubins were within the normal ranges. His albumin level was 29 g/L. His prothrombin time was 13.6 seconds (normal control 12 seconds). Three sets of blood cultures had no growth up to 72 hours of incubation. The patient’s urine culture was sterile. His cerebrospinal fluid was sterile with no cells. A 2D echocardiogram did not show any vegetations. The result of a chest radiograph was normal. The result of the Mantoux test was negative. The result of an ultrasound scan of the patient’s abdomen was negative for organomegaly, free fluid, or intraabdominal lymphadenopathy. An ultrasound scan of his scalp showed a soft tissue mass lesion measuring 3.7 × 3.5 cm in the subgaleal region and no extension into the skull. Non-contrast-enhanced computed tomography (CT) of the brain performed with the bone window revealed soft tissue density outside the skull without intracranial extension.

**Fig. 1:**
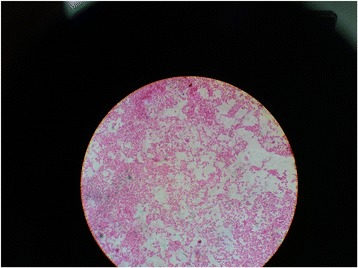
Gram negative bacilli were seen in the gram stain with bipolar staining resembling safety pin appearance

**Fig. 2:**
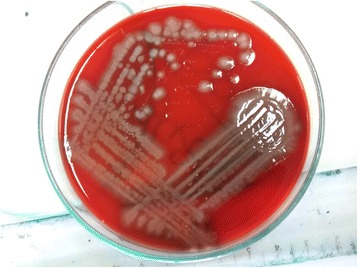
Non fermenting colonies with metallic sheen were isolated on Blood agar and Mac Conkey agarNon fermenting colonies with metallic sheen were isolated on Blood agar and Mac Conkey agar

Surgical exploration was done with the patient under general anesthesia, and pus collection was noted in the subaponeurotic space of the scalp. The pus was drained, and the wound was kept open. Gram-negative bacilli were seen in the Gram stain with bipolar staining resembling a safety pin appearance (Fig. 1). Nonfermenting colonies with a metallic sheen were isolated on blood agar and MacConkey agar (Fig. 2). *B. pseudomallei* was isolated in the culture by the microbiology department of the University of Colombo using the latex agglutination test. The VITEK 2 automated identification system isolated *B. pseudomallei* with 90% probability. The results of an indirect hemagglutination assay for melioidosis antibodies were highly positive with a titer > 10,240. The results of contrast-enhanced CT of the patient’s chest, abdomen, and pelvis were negative for occult abscess.

A diagnosis of superficial melioidosis infection was made, and the patient was started on intravenous ceftazidime 2 g 6-hourly, which was continued for 14 days. The patient was started on eradication therapy with oral co-trimoxazole 960 mg 12-hourly after the intensive phase and continued for 3 months. He was clinically improving and was fever-free by day 5 of treatment, and he was discharged on day 14 of treatment with ceftazidime. On discharge, the patient’s ESR was 45 mm in the first hour, and his CRP level was 10 u/L. He was followed in the clinic and was completely asymptomatic with normal inflammatory markers at 3 months.

## Discussion

PUO, first described by Petersdorf and Beeson [1], is among the most difficult diagnostic problems encountered in internal medicine. Petersdorf and Beeson defined PUO as temperatures > 38.3 °C (> 101 °F) on several occasions, a duration of fever > 3 weeks, and failure to reach a diagnosis despite 1 week of inpatient investigation [1]. Melioidosis is a rare but emerging infection that can present as PUO.

Melioidosis is an infectious disease of humans and animals caused by *B. pseudomallei*. It is a gram-negative, bipolar, aerobic, motile, rod-shaped bacterium found in soil and water [2]. The organism was identified by Whitmore and Krishnaswami in Rangoon, Burma, in 1911 [3]. Melioidosis is endemic in parts of Southeast Asia, but it has increasingly been reported in Sri Lanka during the last decade. The presentation varies from an acute fulminant septicemia to a chronic, debilitating, localized infection and abscess formation [4]. It affects individuals with contact with soil or water, mainly in the rainy season [5]. The bacteria reach the body by inhalation or ingestion of contaminated water or food.

Although healthy individuals can acquire melioidosis, the major risk factors include diabetes, heavy alcohol use, chronic pulmonary disease, chronic renal disease, thalassemia, glucocorticoid therapy, and cancer [6]. Our patient was a paddy farmer from North Central Province of Sri Lanka, had exposure to soil, and had had poorly controlled type 2 diabetes mellitus for 10 years.

Melioidosis has a wide range of signs and symptoms that can be mistaken for other diseases, such as tuberculosis or more common forms of pneumonia. It can present as localized infections, pulmonary infection, bloodstream infection, or disseminated infection [7]. There can be very atypical presentations, such as liver and spleen involvement [8, 9], osteomyelitis [10], septic arthritis [11], and lymphadenitis [12]. Localized infection with suppuration leading to abscess formation is a common presentation of melioidosis. Melioidosis can present as superficial or deep-seated abscesses [13]. Authors of a recent review of case reports in Malaysia described isolation of melioidosis, in descending frequency, in abscesses involving the subcutaneous tissue, liver, spleen, lung, prostate, mycotic pseudoaneurysms, spinal cord, and parotid glands [14].

Our patient had been systemically unwell for 1 month with high inflammatory markers upon presentation, but the only finding of his clinical examination was a small, boggy swelling of the back of the scalp that had been neglected as the cause of sepsis by many treating doctors. The differential diagnosis of surgeons upon clinical examination of the lump was an infected sebaceous cyst or a lipoma, because the patient’s swelling lacked most features of acute inflammation and suppuration. Surgical exploration of the lump revealed pus in an anatomically unusual tissue plane: the subgaleal space. Subgaleal abscesses are rarely encountered today [15]. Usually, subgaleal abscesses develop after trauma or puncture wounds [16–18]. In the literature, we found only one case report of a *de novo* subgaleal abscess; it occurred in a 62-year-old woman without antecedent trauma or injury, and surgical drainage of it yielded a purulent exudate with a pure growth of *Streptococcus pyogenes* [19]. This is the second case of *de novo* subgaleal abscess reported. The predominant organism isolated from posttraumatic and postsurgical scalp infections is *Staphylococcus aureus* [19]. However, other organisms, such as *S. pyogenes* [20] and *Eikenella corrodens* [21], have been reported. However, *B. pseudomallei* has never been reported as the causative organism.

Diagnosis of melioidosis may be challenging for the treating physician. Isolation of *B. pseudomallei* in culture is essential to confirming the initial clinical diagnosis. *B. pseudomallei* grows in blood agar and MacConkey agar, and it can be misidentified as a *Pseudomonas* species [22]. A widely used selective medium for isolating *B. pseudomallei* is Ashdown’s medium, which is not available in Sri Lanka [23]. In our patient’s case, cultures with routine media showed a gram-negative rod that had bipolar staining resembling a safety pin. A strong suspicion of melioidosis was raised on the basis of the atypical location of the abscess, patient risk factors, and culture appearance that led us to actively screen for the organism. Species identification in our patient was done using the VITEK 2 automated identification system and the latex agglutination test. Nucleic acid amplification tests are available for molecular identification of *B. pseudomallei.* There are different serodiagnostic methods available for the identification of the strain. The tests include enzyme-linked immunosorbent assays and indirect hemagglutination assays [24].

Treatment has two phases: (1) the intravenous intensive phase for acute disease, followed by (2) the eradication phase. Intravenous ceftazidime (2 g 6-hourly) and meropenem (1 g 8-hourly) are agents of choice in the intensive phase. Co-trimoxazole is used in the eradication phase [25]. The duration of treatment depends on local versus systemic infection. Our patient had a subgaleal abscess. He was extensively investigated to look for deep-seated occult foci of melioidosis, including CT of the brain and contrast-enhanced CT of the chest, abdomen, and pelvis, the results of which were negative. The diagnosis of superficial localized melioidosis infection was made, and an intensive treatment phase with ceftazidime for 2 weeks was initiated, followed by an eradication phase with co-trimoxazole for 3 months.

## Conclusions

Melioidosis is an emerging infection in Sri Lanka that can present as PUO. It can be serious and life-threatening. Its clinical presentation is diverse. Abscesses in anatomically atypical locations should alarm the treating physician of the possibility of melioidosis. Diabetes is an important risk factor. Special microbiological investigations are needed in suspected cases to confirm the diagnosis, and prolonged treatment with antimicrobials is needed. With prompt diagnosis and timely treatment, the prognosis of patients with melioidosis is excellent.
